# Three-dimensional vertical structural electrochemical random access memory for high-density integrated synapse device

**DOI:** 10.1038/s41598-023-41202-5

**Published:** 2023-08-31

**Authors:** Hyejin Kim, Jongseon Seo, Seojin Cho, Seonuk Jeon, Jiyong Woo, Daeseok Lee

**Affiliations:** 1 Department of Electronic Materials Engineering, Kwangwoon University, Seoul, 01897 Republic of Korea; 2 School of Electronic and Electrical Engineering, Kyungpook National University, Daegu, 41566 South Korea

**Keywords:** Electrical and electronic engineering, Electronic devices, Information storage

## Abstract

Three-terminal (3T) structured electrochemical random access memory (ECRAM) has been proposed as a synaptic device based on improved synaptic characteristics. However, the proposed 3T ECRAM has a larger area requirement than 2T synaptic devices; thereby limiting integration density. To overcome this limitation, this study presents the development of a high-density vertical structure for the 3T ECRAM. In addition, complementary metal-oxide semiconductor (CMOS)-compatible materials and 8-inch wafer-based CMOS fabrication processes were utilized to verify the feasibility of mass production. The achievements of this work demonstrate the potential for high-density integration and mass production of 3T ECRAM devices.

## Introduction

Recently, unstructured data such as videos, images, sounds, and portable document format files have been growing at an exponential rate, leading to a significant increase in the amount of data that needs to be processed^1^. The efficient processing of such vast amounts of data requires faster data processing with low-power consumption. However, the conventional Von-Neumann architecture, which processes data via a series of operations between the central processing unit and memory unit, has a bottleneck effect that causes slow data processing^2–5^. To overcome this, researchers have explored neuromorphic computing systems utilizing parallel data processing, which enables faster and more energy-efficient processing of humungous amounts of data^6,7^. To implement such neuromorphic computing systems in hardware, each synaptic device in the array must satisfy several requirements, such as linear and symmetric conductance modulation under identical pulse bias, wide on/off conductance ratio, data retention, and endurance. Based on these requirements, precise and energy-efficient computing processes (involving training and inference) can be achieved^8^. Previous studies have extensively investigated various simple and high-density two-terminal (2T) memory devices, such as resistive random access memory^9^, phase-change memory^10^, and ferroelectric random access memory^11^. However, the previously proposed 2T synaptic devices exhibit non-ideal synaptic characteristics that degrade system accuracy, including abrupt conductance changes, small on/off ratio, sneak current issues in arrays, and reliability issues (data retention, and endurance)^12–15^.

To address these limitations, three terminals (3T) structured synaptic devices have been proposed, particularly the ion-based electrochemical random access memory (ECRAM), which operates via ionic electrochemical reactions. Compared to 2T synaptic devices, 3T ECRAMs exhibit significantly improved synaptic characteristics, such as low-power operation, larger on/off ratio, linear conductance modulation, more reliable retention, and excellent endurance^16,17^. Among the ECRAMs with various ions, oxygen ion based ECRAMs (OxECRAMs) have the added advantage of using CMOS fabrication processes^18,19^. Despite the improved synaptic characteristics exhibited by OxECRAM, there is still an obstacle in terms of the integration density. Compared to 2T synaptic devices, the 3T OxECRAM requires a larger area owing to its 3T structure. Therefore, in this research, we propose a vertical structure for the 3T OxECRAM (V-ECRAM), which can overcome the limitation in integration density. We utilized 8-inch wafer-based CMOS fabrication processes through a semiconductor fab to verify the feasibility of mass production.

## Methods

The V-ECRAM was fabricated using an 8-inch wafer-based CMOS fabrication process. Ti/TiN/Ti/SiO$_2$/Ti/TiN /SiO$_2$ stacks were formed on a 1,000 nm thick thermally grown SiO$_2$. Both Ti and TiN layers were deposited using a sputtering system while the SiO$_2$ layer was formed via plasma-enhanced chemical vapor deposition. The two TiN layers play the role of the source and drain electrodes, respectively. The Ti layers were deposited to serve as an adhesion layer for the TiN and SiO$_2$ layers. Subsequently, it was etched in the shape of a via-hole with 1.5 $\mu$m diameter. Finally, the channel/electrolyte/oxygen reservoir/gate electrode were deposited on the side wall of the via-hole.

**Figure 1:**
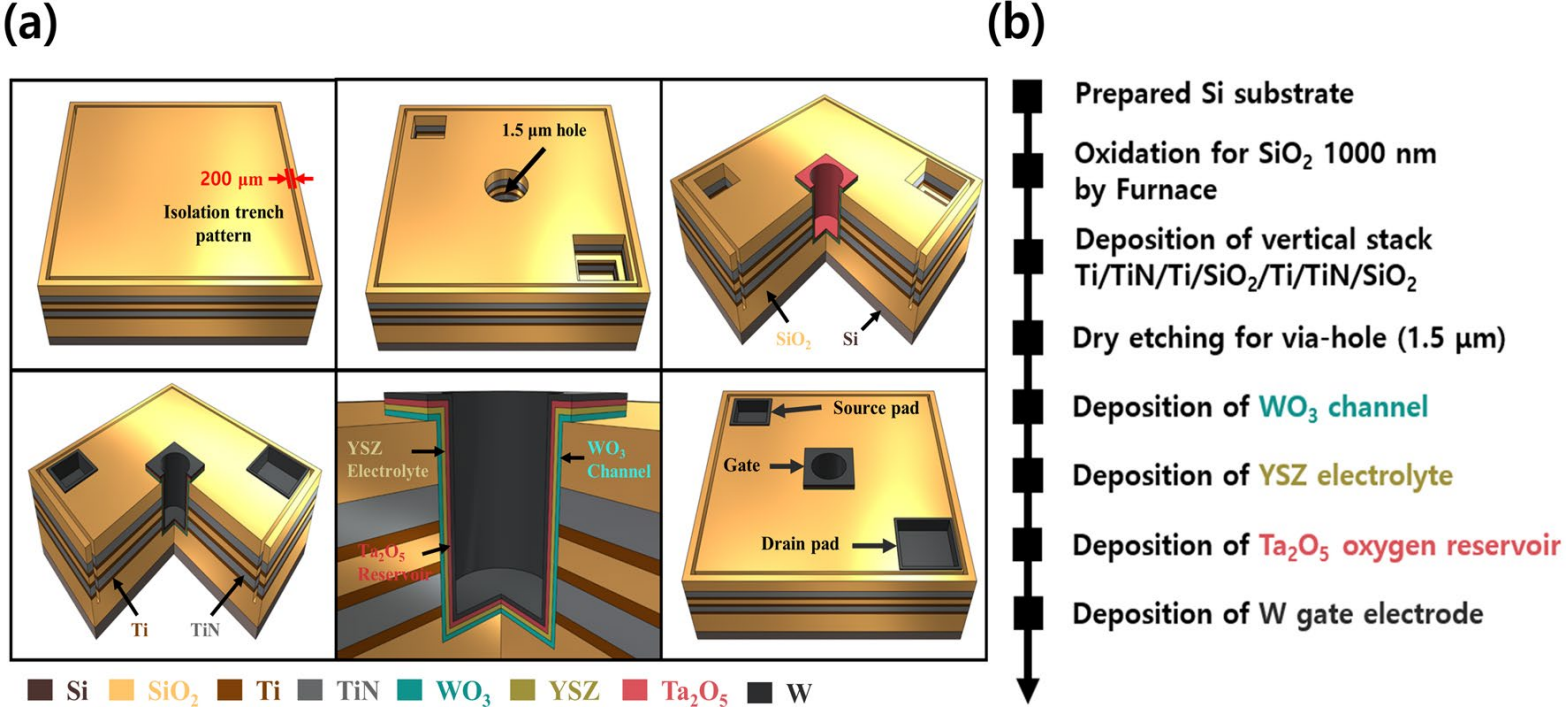
(**a**) Fabrication schematic image and (**b**) flow diagram of V-ECRAM manufactured using a three-dimensional vertical structure wafer.

For all cases, a WO$_3$ (60 nm) channel was deposited via reactive sputtering using a WO$_3$ target in Ar and O$_2$ mixed ambient gas. Thereafter, an approximately 3 nm Yttria-stabilized zirconia (YSZ) electrolyte and approximately 230 nm of Ta$_2$O$_5$ oxygen reservoir were deposited in Ar ambient gas. Lastly, W layer (50 nm) was deposited as the gate electrode in an Ar ambient gas. The WO$_3$ and YSZ were deposited at working pressures of 5 mTorr and 40 mTorr, respectively. Moreover, Ta$_2$O$_5$ and W were deposited at 10 mTorr. The fabrication schematic and flow diagram are depicted in Fig. 1a,b.

Electrical analyses were conducted using a semiconductor parameter analyzer (HP 4156A) and pulse generator (Agilent 81110A). A series of positive and negative identical pulses were applied to the gate electrode and read operation was conducted on the source-drain electrode by applying read bias. During the writing process, all electrical measurements were performed with a common ground for both the source and drain, while applying gate bias. During the reading process, only the source was grounded, a read bias (V$_{SD}$ = 0.5 V) was applied to the drain. For each sample, all the conditions during fabrication and electrical analyses were optimized to achieve the best synaptic characteristics.

## Results and Discussion

**Figure 2:**
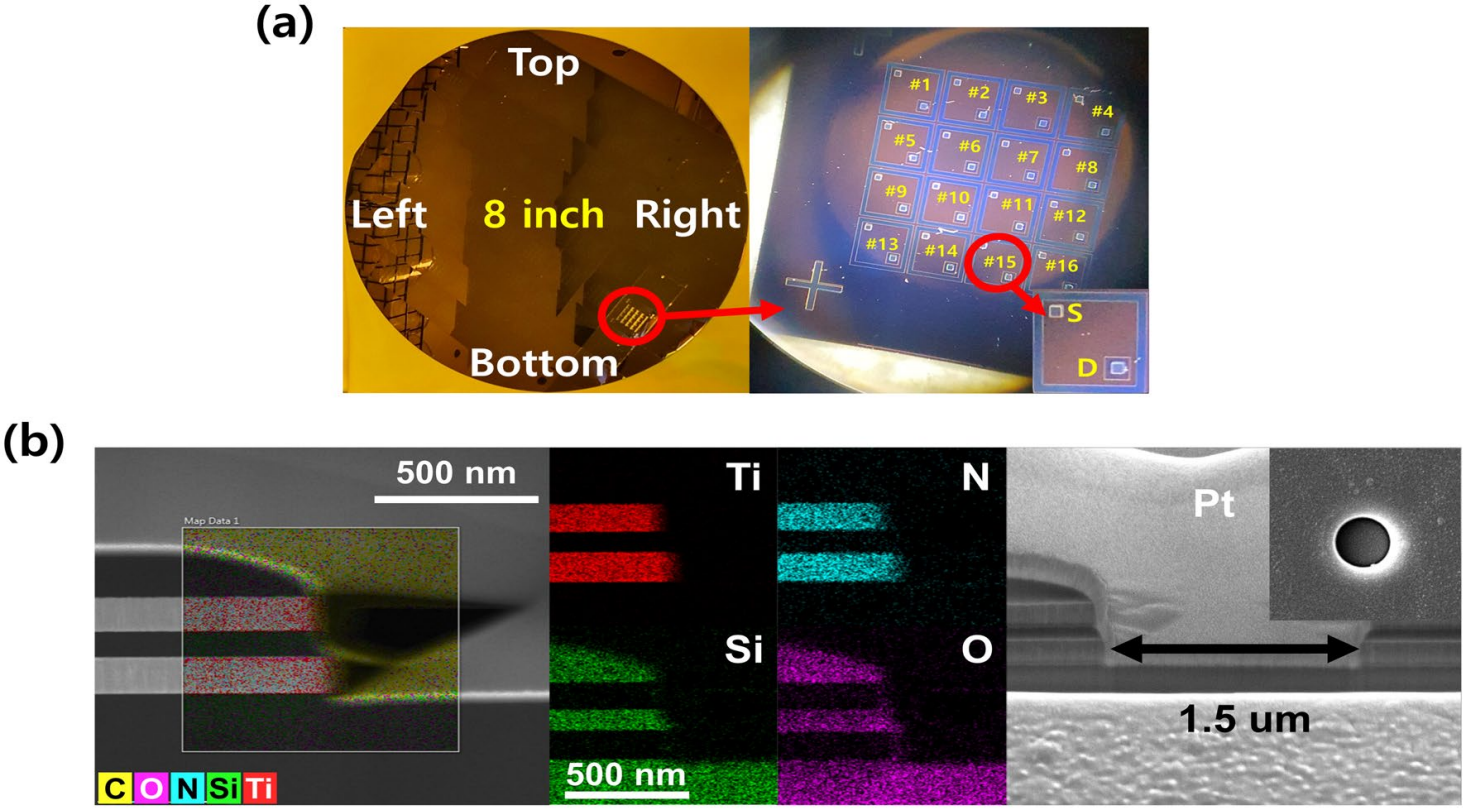
(**a**) 8-inch wafer with 3D vertical structure for high-density and mass production. There are $4 \times 4$ cells (total 16 cells) in one die. (**b**) EDS mapping and cross-sectional TEM image of 3D vertical structure. The results show that Ti/TiN/Ti/SiO$_2$/Ti/TiN/SiO$_2$ stacks were properly deposited on the thermally grown SiO$_2$ substrate.

In this study, we propose a 3D vertical structure to overcome the limitations in integration density of 3T OxECRAM. Compared to the existing 3T-based ECRAMs, which occupy more than 10F$$^2$$ of a single cell^20,21^, the proposed V-ECRAM has the advantage of being reduced to 4F$$^2$$ because the source and drain are vertically stacked. To realize this, the 3D vertical structure was fabricated using an 8-inch fabrication process (Fig. 2a). Figure 2b shows the transmission electron microscope (TEM) image and energy dispersive x-ray spectrometer (EDS) mapping of the fabricated 3D vertical structure. These results confirm that $$_2$$$$_2$$ Ti/TiN/Ti/SiO$$_2$$/Ti/TiN/SiO$$_2$$stacks were properly deposited on the thermally grown SiO$$_2$$ substrate with a thickness of 1,000 nm. The WO$$_3$$, YSZ, Ta$$_2$$O$$_5$$, and W of CMOS compatible materials were sequentially deposited as channel, electrolyte, oxygen reservoir, and gate electrode on the side wall of via-hole with 1.5 $$\mu$$m diameter formed via typical dry etching. The device manufacturing process is described in detail in Fig. 1 and the “Methods” Section. The possibility of high-density integration and mass production of the synapse device was confirmed by successfully fabricating a 3T (gate, source, and drain)-based V-ECRAM.

The operation mechanism of V-ECRAM is the same as that of the oxygen based ECRAM reported so far^22,23^. When positive bias is applied to the gate electrode, oxygen ions de-doped from the channel layer migrated the oxygen reservoir through the electrolyte layer, increasing the conductance of the channel layer (potentiation process). On the contrary, when negative bias is applied, oxygen ions are doped from the oxygen reservoir to the channel layer, resulting in a depression process in which the conductance of the channel layer is reduced. Based on this operation mechanism, the electrolyte should act as an ionic path between the channel and the oxygen reservoir layer to facilitate the movement of oxygen ions^20,24^. Therefore, we used YSZ with excellent ionic conductivity as the electrolyte^19^, and the YSZ layer was controlled to optimize device performance.

**Figure 3:**
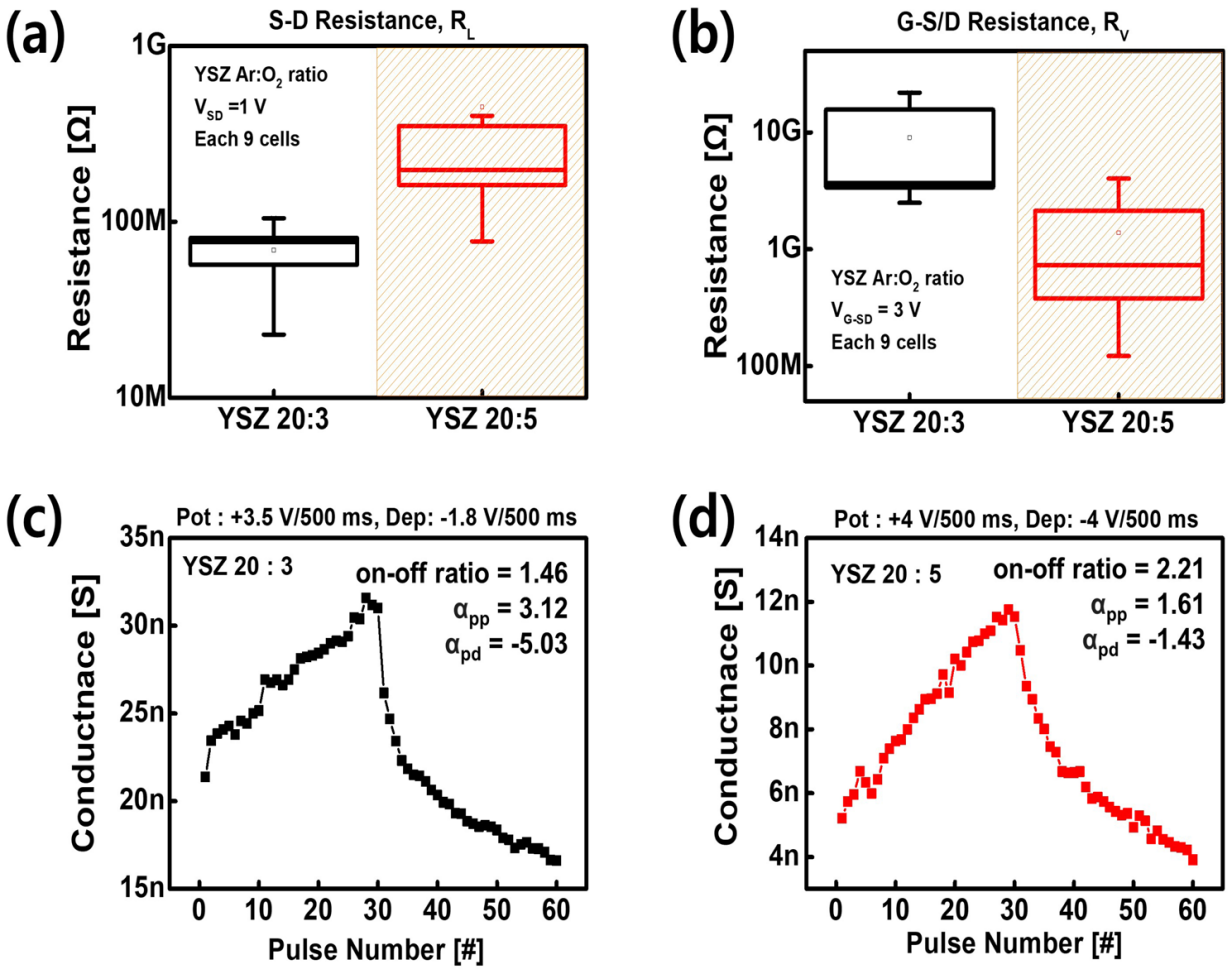
Nine cells distribution of (**a**) lateral (source-drain) and (**b**) vertical (gate-source/drain) resistance. The potentiation-depression of (**c**) YSZ 20:3 and (**d**) YSZ 20:5 for synaptic characteristic.

The ratio of Ar and O$_2$ mixed ambient gas was controlled to optimize the YSZ electrolyte layer. Figure 3 presents a comparison of the characteristics of Ar: O$_2$ = 20 sccm: 3 sccm and 20 sccm: 5 sccm, which were named YSZ 20:3 and YSZ 20:5, respectively. The source-drain (lateral resistance, R$_L$) and gate-source/drain resistance (vertical resistance, R$_V$) in the initial state, which varies with the O$_2$ ratio, are shown in Fig. 3a,b. Thus, as the O$_2$ ratio of YSZ increases, R$_L$ increases and R$_V$ decreases. For R$_L$, it can be explained that oxygen ions move to a more stable material owing to the difference in Gibbs free energy between the WO$_3$ channel and the YSZ electrolyte during the deposition of YSZ layer. The Gibbs free energy for oxide formation of each material is as follows: YSZ (-1755.26 kJ/mol) < WO$_3$ (-756.7 kJ/mol)^25,26^. Consequently, oxygen ions de-doped from the channel layer during YSZ electrolyte deposition are absorbed into the YSZ electrolyte layer with a more stable state. When the O$_2$ ratio increases from YSZ 20:3 to 20:5, the oxygen vacancies in the YSZ layer decrease during the deposition of the YSZ layer, resulting in a relative decrease in the amount of oxygen ions absorbed from the WO$_3$ channel layer to the YSZ electrolyte layer. Accordingly, in the case of YSZ 20:5, the oxygen ions in the WO$_3$ channel layer are larger than those of YSZ 20:3, which increases R$_L$, which is the resistance between the source and drain (Fig. 3a).

In contrast, R$_V$ decrease as the O$_2$ ratio increases, primarily because YSZ has the characteristics of a p-type semiconductor^27^. Therefore, as the O$_2$ ratio increases from YSZ 20:3 to 20:5, the resistance of the YSZ layer decreases, reducing the overall R$_V$ (Fig. 3b). Both YSZ 20:3 and 20:5 have significantly higher R$_V$ than R$_L$. In other words, the channel conductance can be evaluated without the effect of gate leakage when reading the channel conductance.

The potentiation and depression were measured to evaluate the synaptic characteristics of the optimized device. Once a pulse was applied to the gate electrode, a read voltage (V$_{SD}$ = 0.5 V) was applied to the source and drain to read the conductance of the channel. The gate pulse amplitude and width of each device are +3.5 V/-1.8 V, 500 ms for YSZ 20:3 and +4 V/-4 V, 500 ms for YSZ 20:5, which is optimized for each device. Furthermore, the linearity parameter ($\alpha$) was setted to evaluate the linearity of the weight-update curve, as follows.

1 $$\begin{aligned}{} & {} G = {\left\{ \begin{array}{ll} ((\textrm{G}_{MAX}^{\alpha }-{G}_{MIN}^{\alpha })\times \omega + {G}_{MIN}^{\alpha })^{\frac{1}{\alpha }}) &{} \text {if} \,\, \alpha \ne 0, \\ {G}_{MIN} \times ({G}_{MAX}/{G}_{MIN})^{\omega } &{} \text {if} \,\, \alpha = 0. \end{array}\right. } \end{aligned}$$

2 $$\begin{aligned}{} & {} \text {G}_{MAX}/G_{MIN} \end{aligned}$$

Where, G$_{MAX}$ and G$_{MIN}$ are maximum conductance state and minimum conductance state, respectively, and $\omega$ is an internal variable which ranges from 0 to 1. The ideal case is when $\alpha$ = 1. Based on Equation (1), the linearity of potentiation ($\alpha _{pp}$) and depression ($\alpha _{pd}$) was calculated. As a result of evaluating the linearity parameter according to the Ar:O$_2$ ratio, the $\alpha$ $_{pp}$ and $\alpha _{pd}$ of the YSZ 20:5 is 1.61, -1.43, respectively. In addition, the on/off ratio was obtained based on Equation (2). The on/off ratio of the YSZ 20:5 is 2.21, which exhibits a more improved synaptic characteristics than YSZ 20:3 (Fig. 3c,d).

The relatively lower vertical resistance of the YSZ 20:5 is the reason for the better performance compared to the YSZ 20:3. The voltage drop across the YSZ layer is relatively small in YSZ 20:5. Therefore, the electric field applied to the channel is larger. Consequently, the migration of doped and de-doped oxygen ions in the channel becomes more active and exhibits stable characteristics. Furthermore, the porosity of the YSZ layer increases when the Ar:O$_2$ ratio increases in the process of deposition of the YSZ layer^28^. Therefore, in YSZ 20:5, the migration path of the oxygen ions is formed to facilitate the ion movement, and this leads to increase the ion mobility^29^.As a result, YSZ 20:5 has a more linear and symmetrical conductance change in the channel layer and a larger on/off ratio, so that improved synaptic characteristics can be achieved. However, the on/off ratio is still one of the importatnt synaptic characteristics to be improved. To overcome this limitation, there is a method of reducing the thickness of the gate and channel layer. By reducing the thickness between the gate and channel layer, a stronger electric field will be applied to the channel when the gate bias is applied. This leads to increase the ion movement, thereby increasing the on/off ratio^20^.

**Figure 4:**
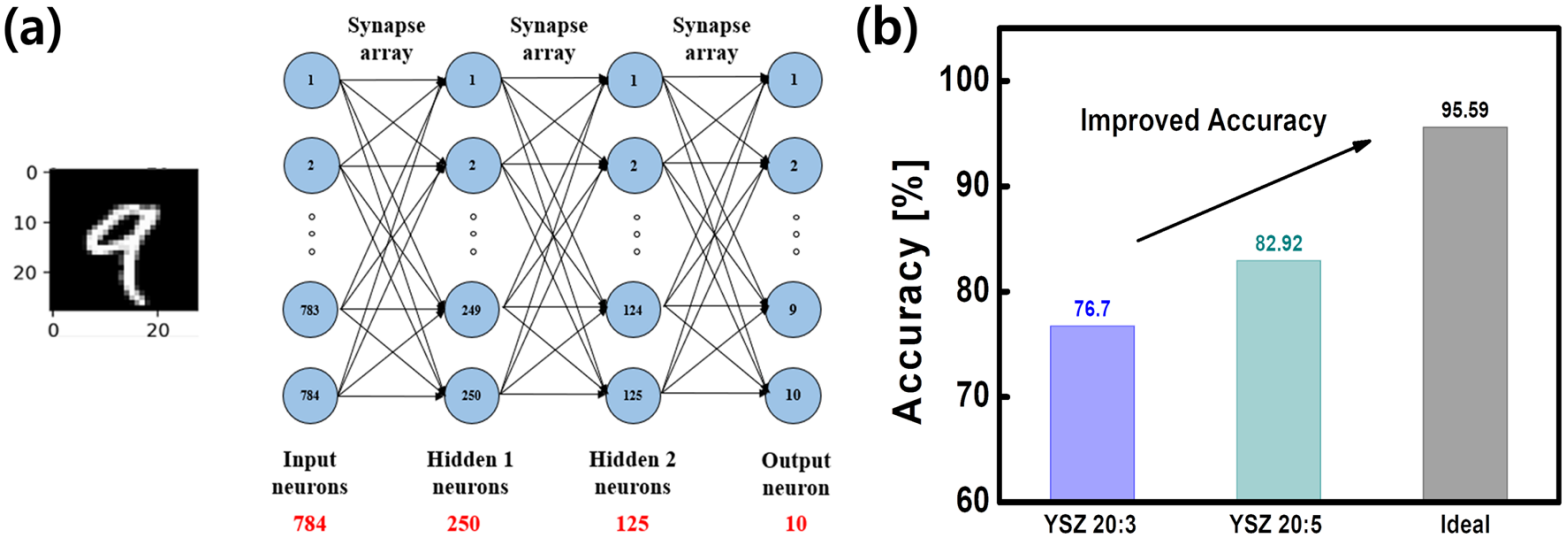
MNIST pattern recognition accuracy of the YSZ 20:3 and YSZ 20:5. (**a**) Schematic of four-layer neural network. MNIST dataset pattern recognition accuracy was evaluated by 784 inputs $\times \,250$ first hidden $\times$ 125 second hidden $\times$ 10 output neurons. (**b**) Pattern recognition accuracy for YSZ 20:3, YSZ 20:5, and ideal case.

A pattern recognition simulation comprising four-layers was performed to verify the system-level performance of the V-ECRAM. The neural network was constructed with an input layer (784 neuron nodes), hidden layer 1 (250 neuron nodes), hidden layer 2 (125 neuron nodes), and output layer (10 neuron nodes) (Fig. 4a). The neuron node of each layer was connected to the next neuron node via a synapse device. We used the Modified National Institute of Standards and Technology (MNIST) dataset (28 $\times$ 28) as an input image. To compare the accuracy, potentiation/depression linearity, on/off ratio, and number of multilevel of V-ECRAM were considered in the simulation.

The results confirm that the vertical 3T synapse device fabricated using 8-inch wafer-based CMOS fabrication demonstrates the feasibility of high-density integration and mass production. We improved the synaptic characteristics, such as linearity of the potentiation/depression and on/off ratio by optimizing the YSZ electrolyte layer, which plays a crucial role in the ECRAM device. Accordingly, in Fig. 4b, we confirmed that the pattern recognition accuracy at the system-level was improved from 76.70% to 82.92%. Although the synaptic characteristics of synaptic devices have been secured, they still need to be improved compared to ideal synaptic characteristics^30^. In addition, research on reliability characteristics such as retention and endurance are also essential to continuously operate the device.

## Conclusion

The V-ECRAM was successfully developed using an 8-inch wafer-based CMOS fabrication process. Therefore, the area occupied by single cell was reduced from 10F$^2$ to 4F$^2$, demonstrating the feasibility of high-density integration and mass production. From the optimization of electrolyte layer, in Table 1, improved synaptic characteristics such as linearity of the potentiation/depression (1.61/–1.43) and on/off ratio (2.21) were achieved, which resulted in improved recognition accuracy from 76.70% to 82.92%. These results obviously demonstrate the feasibility of mass production and high integration density of the 3T OxECRAM device.

**Table 1:** Comparison of the 2T, planar 3T, and vertical 3T (this work) synaptic device. The V-ECRAM demonstrated the feasibility of high-density integration and mass production.

	Reference^31^	Reference^32^	This work
Type	Two-terminal	Two-terminal	Three-terminal
Structure	Planar	Planar	Vertical
Dimension	4F$^2$	10$^2$	4F$^2$
Channel length	1 $\mu$m	100 $\mu$m	0.1 $\mu$m
Linearity (pot./dep.)	–	0.69/0.42	1.61/–1.43
On/off ratio	2.25	2.17	2.21
